# Enhancing Medication Safety through Implementing the Qatar Tool for Reducing Inappropriate Medication (QTRIM) in Ambulatory Older Adults

**DOI:** 10.3390/healthcare12121186

**Published:** 2024-06-12

**Authors:** Ameena Alyazeedi, Mohamed Sherbash, Ahmed Fouad Algendy, Carrie Stewart, Roy L. Soiza, Moza Alhail, Abdulaziz Aldarwish, Derek Stewart, Ahmed Awaisu, Cristin Ryan, Phyo Kyaw Myint

**Affiliations:** 1 Pharmacy Department, Rumailah Hospital, Hamad Medical Corporation, Doha P.O. Box 3050, Qatar; msherbash@hamad.qa (M.S.); amohamed89@hamad.qa (A.F.A.); 2 Ageing Clinical & Experimental Research (ACER) Team, Institute of Applied Health Sciences, University of Aberdeen, Foresterhill, Aberdeen AB25 2ZD, Scotland, UK; carrie.stewart@abdn.ac.uk (C.S.); roy.soiza@nhs.net (R.L.S.); phyo.myint@abdn.ac.uk (P.K.M.); 3 NHS Grampian, Aberdeen Royal Infirmary, Aberdeen University, Aberdeen AB25 2ZN, Scotland, UK; 4 Pharmacy Executive Director’s Office, Hamad Medical Corporation, Doha P.O. Box 3050, Qatar; malhail2@hamad.qa; 5 Rumailah Administration, Hamad Medical Corporation, Doha P.O. Box 3050, Qatar; adarwish1@hamad.qa; 6 Department of Clinical Pharmacy and Practice, College of Pharmacy, QU Health, Qatar University, Doha P.O. Box 2713, Qatar; d.stewart@qu.edu.qa (D.S.); aawaisu@qu.edu.qa (A.A.); 7 School of Pharmacy and Pharmaceutical Sciences, Trinity College Dublin, D02 PN40 Dublin, Ireland; cristin.ryan@tcd.ie

**Keywords:** older adults, deprescribing, pharmacist intervention, polypharmacy, potentially inappropriate medication (PIM), medication management, key performance indicators (KPIs), quality improvement

## Abstract

Introduction: To promote optimal healthcare delivery, safeguarding older adults from the risks associated with inappropriate medication use is paramount. Objective: This study aims to evaluate the effectiveness of implementing the Qatar Tool for Reducing Inappropriate Medication (QTRIM) in ambulatory older adults to enhance medication safety. Method: The QTRIM was developed by an expert consensus panel using the Beers Criteria and contained a list of potentially inappropriate medications (PIMs) based on the local formulary. Using quality improvement methodology, it was piloted and implemented in two outpatient pharmacy settings serving geriatric medicine and dermatology clinics at Rumailah Hospital, Qatar. Key performance indicators (KPIs) using implementation documentation as a process measure and the percentage reduction in PIM prescriptions as an outcome measure were assessed before and after QTRIM implementation. This study was conducted between July 2022 and September 2023. Results: In the outpatient department (OPD) geriatric pharmacy, the prescription rate of PIMs was reduced from an average of 1.2 ± 0.7 PIMs per 1000 orders in 2022 to an average of 0.8 ± 0.2 PIMs per 1000 orders in 2023. In the OPD geriatric pharmacy, the results showed a 66.6% reduction in tricyclic antidepressants (TCAs) (from 30 to 10), a reduction in first-generation antihistamines by 51.7% (29 to 14), and muscle relaxants by 33.3% (36 to 24). While in dermatology, the older adult prescription rate of PIMs was reduced from an average of 8 ± 3 PIMs per 1000 orders in 2022 to a rate of 5 ± 3 PIMs per 1000 orders in 2023; the most PIM reductions were (49.4%) in antihistamines (from 89 to 45), while muscle relaxants and TCAs showed a minimal reduction. Conclusions: Implementing QTRIM with pharmacy documentation monitoring markedly reduced the PIMs dispensed from two specialized outpatient pharmacies serving older adults. It may be a promising effective strategy to enhance medication safety in outpatient pharmacy settings.

## 1. Plain Language Summary

This study focused on improving the safety of medication prescribing while dispensing in outpatient pharmacies by implementing the Qatar Tool for Reducing Inappropriate Medication (QTRIM) and monitoring key performance indicators (KPIs). The goal was to apply QTRIM to enhance medication safety by improving dispensing accuracy through reducing PIM. This study, conducted in two OPD pharmacies at Rumailah Hospital in Qatar, spans from July 2022 to September 2023 and evaluates the implementation of QTRIM in outpatient geriatric medicine and dermatology pharmacy services. By selecting these specific services, this research aims to address the unique needs of different patient groups.

This study’s findings showed reduced potentially inappropriate medications (PIMs). In the outpatient geriatric pharmacy, the results show that TCAs were reduced by two-thirds, first-generation antihistamines by half, and muscle relaxants by less than one-third. In dermatology, first-generation antihistamine prescriptions had a half reduction in prescribing, while muscle relaxants and TCAs showed a minimal reduction. This reduction in PIM dispensing in the OPD setting provides valuable insights into how QTRIM and pharmacy monitoring KPIs affect medication dispensing accuracy. This study is expected to be a foundational resource for future initiatives enhancing medication safety in outpatient pharmacy environments.

In conclusion, this study suggests integrating QTRIM and strategically using pharmacy monitoring KPIs to improve medication safety. The findings are expected to contribute significantly to discussions on optimizing medication management protocols, offering guidance for healthcare institutions looking to strengthen patient safety through proactive quality improvement measures.

## 2. Introduction

Aging and associated changes in physiology are inevitable and bring unique healthcare challenges. As a result, medication management for older people becomes complex and problematic, and various efforts have been made to optimize this, primarily by trying to avoid drugs deemed to be inappropriate [1]. It is because this stage of life also represents specific challenges related to medication use, including polypharmacy coupled with an increase in Potentially Inappropriate Medications (PIMs), poor medication adherence, and potential drug interactions [2].

The concept of ‘appropriate polypharmacy’ suggests that patients can benefit from multiple evidence-based medications while ‘inappropriate polypharmacy’ should be avoided [2]. Identifying and addressing inappropriate medication use is essential to optimize health outcomes, reduce healthcare costs, and enhance the overall quality of life for the aging population. Several implementation strategies can reduce PIM prescribing [3]. In 2014, a systematic review of the published literature on inappropriate prescribing in frail individuals aged at least 65 found that 20 out of 25 reviewed articles used the Beers Criteria to evaluate inappropriate medication use [4]. In 2023, a systematic review and meta-analysis, including 94 articles, showed that the most used criteria were the Beers Criteria, followed by the STOPP/START criteria; the overall pooled prevalence of PIM was 36.7% for 371.2 million older participants from 17 countries [5].

A Qatari national retrospective study conducted from 2016 to 2018 in the Hamad Medical Corporation (HMC) outpatient setting highlighted a significant association between PIM use, polypharmacy, and adverse events, emphasizing the need for optimized medication management and deprescribing strategies in outpatient settings; it is the only study covering older adults in HMC, and it showed that the prevalence of PIMs was more than 60% [6]. Internationally, efforts have been made to monitor and reduce PIM use in older adults. Examples of these initiatives include the Beers Criteria, WHO Global Patient Safety Challenge, European Union (EU) initiatives, and the Canadian Deprescribing Network [7,8,9,10]. There are limited studies in Qatar on monitoring appropriate and safe medication management in this patient population.

This paper evaluated a quality improvement (QI) tool to promote evidence-based interventions and monitor their impact. This research emphasizes the importance of incorporating proper medication management practices when treating older adults in Qatar.

## 3. Materials and Methods

### 3.1. Study Design and Setting

This quality improvement project was conducted at two outpatient pharmacies within Rumailah Hospital, HMC, in Qatar, between July 2022 and September 2023. This study focused on monitoring Rumailah Hospital outpatient pharmacies’ dispensing practices by applying the intervention tool to reduce dispensing PIMs before and after introducing the intervention tool.

### 3.2. Intervention Development

The Qatar Tool for Reducing Inappropriate Medication (QTRIM) integrated criteria from the globally recognized Beers Criteria for identifying inappropriate prescriptions in older adults, which cannot be used in its original form due to different formulary lists available for safe alternatives, and there are restrictions and approved guidelines for prescribing that should be considered when developing the tool. HMC formulary items from the Beers 2019 [7] list were selected and included in the adapted Beers tool, with certain medications excluded due to their low prevalence of usage. This preliminary list was refined to 30 items of medications that should be avoided in older adult prescribing according to the Beers Criteria and finalized through consultation with a review panel. The review panel included 15 consultants and 7 pharmacists in internal medicine, geriatrics, and mental health to obtain their agreement and feedback. The consultation review panel developed the QTRIM as a starting point (Phase 1) by creating a list of PIMs commonly used that carry higher risk to older adults and including the formulary safe alternatives.

The drafted Qatar Tool for Reducing Inappropriate Medication (QTRIM) list encompassed an HMC formulary list of medications that should be avoided in older adults as per the Beers Criteria, each with safer alternatives. It excluded all medications with concerns or that needed further decisions by other specialties. All feedback and comments were discussed over 3 meetings, and an agreement was reached to include 24 items in Phase 1 as a starting point to be expanded after studying the impact of implementing the new tool.

### 3.3. Implementation Process

Following its development, the QTRIM and the process map for reducing the dispensing PIMs list according to QTRIM were approved by the Rumailah Pharmacy and Therapeutics Committee, and the research was approved by the Medical Research Center’s (MRC) Ethics Committee.

The QTRIM was introduced as a manual tool, as it is not integrated electronically in OPD clinics and pharmacies. It included the safe alternative for each PIM as guidance for prescribers and pharmacists. Pharmacists used the tool while verifying the order and recommending safe alternatives to prescribers if PIMs were prescribed. Monitoring was conducted at the pharmacy level by reviewing all PIM orders by a pharmacist before dispensing. If the PIM order is prescribed, the pharmacist contacts the prescribers to ensure awareness of QTRIM and provides a safe alternative. If the prescriber preferred to prescribe a PIM (instead of its alternative), the pharmacist documented the justification for dispensing the PIM. If the pharmacist could not reach the prescribers, the patient was referred to an MTM or another geriatric clinic if available.

The educational phase for the process map (from July to the end of December 2022) was compared with post-intervention data from January to September 2023.

**Figure 1:**
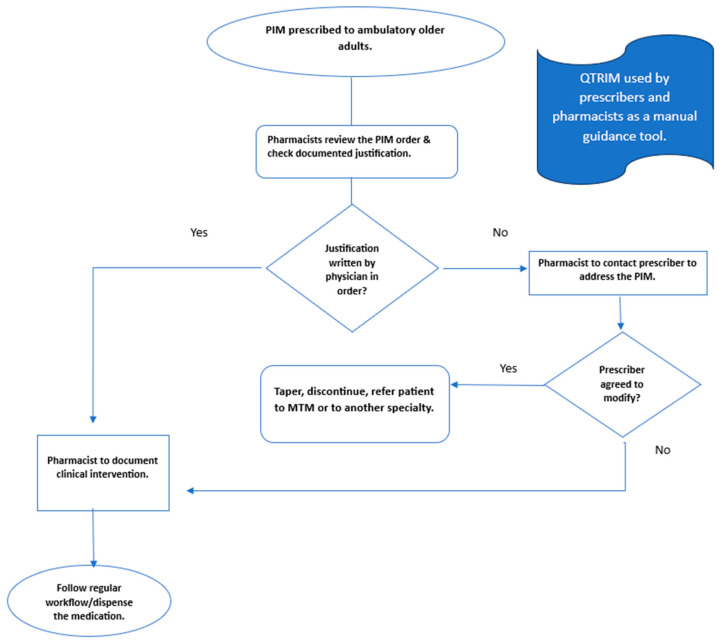
Process map for reducing dispensing PIMs list according to QTRIM. MTM: Medication Therapy Management.

During the education phase, prescribers and pharmacists at the outpatient clinics were trained in the new workflow to ensure seamless integration. This training included familiarizing themselves with the QTRIM list, educating about safer medication alternatives on the list, and facilitating cooperation between prescribers and pharmacists according to the agreed process map presented in Figure 1. The clinicians practicing in the OPD clinics were oriented to the agreed QTRIM workflow, so they would expect phone calls from pharmacists before dispensing the medication if no justification was provided while prescribing the QTRIM.

For (process measure) performance monitoring, pharmacy supervisors reviewed daily automated Excel reports for the previous day’s PIM dispensing and ensured proper documentation was provided for justification. The research team continued to give feedback to pharmacists on their performance after distributing the QTRIM and providing education and training on the agreed process map.

### 3.4. Data Collection and Documentation

The rationale for prescribing PIMs was documented in the Electronic Health Record system by the hospital pharmacist before dispensing by filling out a clinical pharmacist intervention form. Additionally, pharmacy informatics automated a daily morning Excel spreadsheet report of all PIM orders dispensed and relevant pharmacist interventions through the previous day. OP supervisors then reviewed the sheet and confirmed the interventions made by the pharmacists. Staff awareness and performance were monitored in the reported form, and data were documented in a dedicated Excel sheet in a secured shared folder the study team could access.

Statistical analysis: Numerical and categorical data were analyzed descriptively and presented using means (standard deviations) and numbers (percentages). The analysis covered pre- and post-interventional periods. We utilized run charts and rate comparisons to assess the PIM prescription rate in different pharmacy settings. Baseline data, including the educational period from August to December 2022, were compared with post-intervention data from January to September 2023. Additionally, we compared classes of PIMs to detect changes in prescribing patterns of particular drug classes. For a meaningful interpretation, we presented rate measures per 1000 orders in the two pharmacies to allow the comparison of the rates between them. The difference in the rates test along with the percentage reduction in the rates before and after implementation of the intervention will be used to quantify the change magnitude. Percentage reduction = (‘Baseline rate − Post-intervention rate’/Baseline rate) × 100. The *p*-value was considered significant if ≤0.05. All analyses were carried out using Microsoft Excel 365 and STATA 17.0 (Table 1).

### 3.5. Key Performance Indicator (KPI) Measures

Pharmacy monitoring KPIs for performance measures included monitoring the application of the agreed workflow of documenting intervention with the justification of dispensing the PIMs as a process measure and the improvement in reducing the dispensing of a PIM as an outcome measure. The definitions of these measures are presented in Table 2.

## 4. Results

The patients in this study had a mean (SD) age of 71 ± 7.8 years, with 53% being males. The majority of the population was of the Arab race (68%). Table 3 presents the summary results for each period separately and then combined. Three hundred and thirty-seven PIMs were prescribed during the whole study period. The PIMs were 220 in 2022 and reached 117 up to September 2023.

### 4.1. Process Measures (Clinical Interventions Documentation)

#### 4.1.1. Rumailah Hospital Geriatric Outpatient Pharmacy

**Figure 2:**
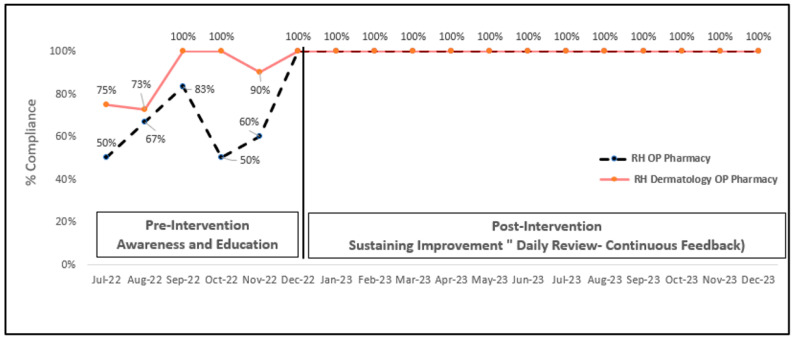
Percentage compliance to clinical intervention documentation in two OP pharmacies and action plan. Individual data are available in Supplementary Tables S1 and S2.

Figure 2 shows different baseline data between the two OP pharmacies, with a lower initial baseline (50% and 75%) for the RH OP pharmacy and OP dermatology pharmacy, respectively. However, both pharmacies reached 100% compliance with pharmacists’ intervention documentation in December 2022. This improvement was sustained throughout the first half of 2023 until the end of 2023.

#### 4.1.2. Challenges Documented in Clinical Intervention

The pharmacists’ clinical documentation showed some challenges faced while reducing the dispensing of PIMs in the OP pharmacy. These challenges were documented by frontline pharmacists while documenting clinical interventions as a process measure.

There were 69 documented clinical interventions with different types of challenges. The main challenge, which constituted approximately 70% of cases (n = 48), was that some PIMs prescribed by psychiatry, neurology, and geriatricians were reluctant to discontinue or reduce PIM doses and insisted on referring the case to the original prescriber. In 20% of cases (n = 14), the patients refused to change their medications despite appropriate counseling from the pharmacist. They argued that they felt well on their current medications, which included PIMs, and hence, they refused to stop any of their medication. The remaining 10% (n = 7) of patients’ prescriptions were from other facilities, such as primary healthcare centers (PHCCs), and their prescribers were not contactable, so the pharmacist was required to refer the patient to MTM or to geriatric consultants to follow the cases.

### 4.2. Outcome Measures

#### RH OP and Dermatology OP PIM Prescription Dispensing Rate/1000 Orders

**Figure 3:**
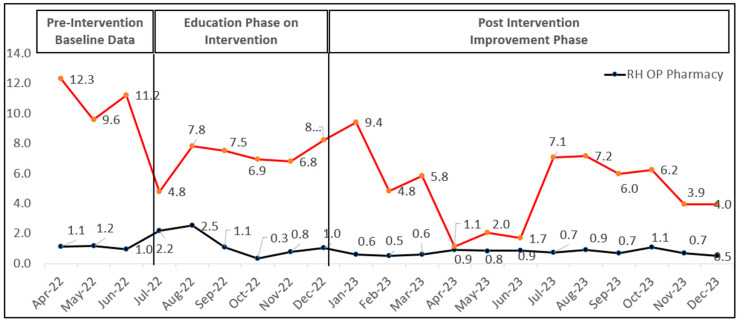
Rate of PIM prescribing in 1000 medication orders in RH OP pharmacy and RH OP dermatology pharmacy. Individual data are available in Supplementary Tables S3 and S4.

Figure 3 shows that, in the RH OP pharmacy, the prescription rate of PIMs was reduced from an average of 1.2 ± 0.7 PIMs per 1000 orders in 2022 to 0.8 ± 0.2 PIMs per 1000 orders in 2023. While in OP dermatology, it was reduced from an average of 8.4 ± 2.3 PIMs per 1000 orders in 2022 to a rate of 5 ± 2.6 PIMs per 1000 orders in 2023. The baseline data included the educational phase up to December 2022, while the post-intervention assessment phase was from January to September. During the improvement phase, in the RH OP pharmacy, the percentage reduction in the PIM rate was 33.3%; however, this was non-significant (*p*-value = 0.26) in 2023 compared to 2022. This is in line with the OP dermatology pharmacy, where there was also a significant percentage reduction in the PIM rate of 41% (*p*-value = 0.002).

### 4.3. Distribution of PIMs by Prescribers Locations

**Figure 4:**
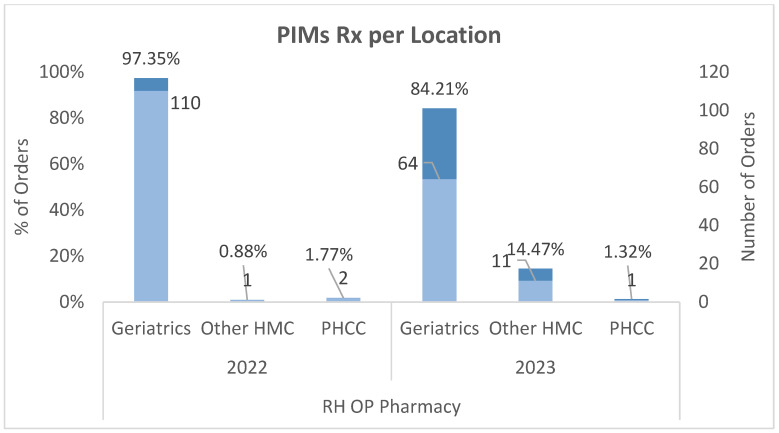
RH OP pharmacy PIM orders by prescriber location.

In RH, in 2022, approximately 97% of all PIM orders were prescribed by geriatricians; however, in 2023, the percentage of geriatricians prescribing PIM orders decreased to 84%, and the percentage of PIM orders prescribed by other HMC specialties and primary healthcare centers (PHCCs) increased from 2.5% in 2022 to around 16% in 2023, as shown in Figure 4.

**Figure 5:**
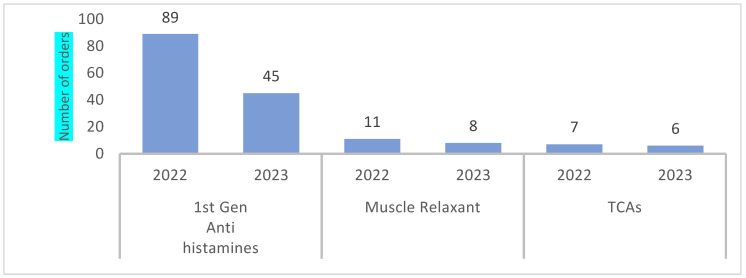
Yearly PIM orders by therapeutic class, RH OPD dermatology pharmacy.

Figure 5 shows the annual prescribing pattern of a specific class of PIMs in the dermatology department from April 2022 to December 2022 compared to January until September 2023. Antihistamine prescriptions had a 49.4% reduction in prescribing frequency from 89 to 45, while muscle relaxants and TCAs showed a minimal reduction.

First-generation antihistamines and muscle relaxants were the most prescribed PIMs across both study years, with a notable decrease in prescriptions over the study period. Process measure compliance with documenting pharmacists’ interventions for justification when identifying and dispensing PIMs showed improvement, reaching 100% compliance toward the end. The PIM prescription rate per 1000 orders decreased (see Figure 3). This study highlighted the prescribing patterns by different departments and specialties, with geriatricians showing a reduction in PIM prescriptions while other specialties slightly increased, as shown in Figure 4.

In the OP geriatric pharmacy, the results show a percentage reduction in TCA, first-generation antihistamines, and muscle relaxants of 66.6% (from 30 to 10), 51.7% (from 29 to 14), and 33.3% (from 36 to 24), respectively. The prescription rate of PIMs was reduced to an average of 1.2 ± 0.7 PIMs per 1000 orders in 2023, compared to an average of 0.8 ± 0.2 PIMs per 1000 orders in 2022. In dermatology, antihistamine prescriptions had a 49.4% reduction in prescribing frequency from 89 to 45, while muscle relaxants and TCAs showed a minimal reduction. The prescription rate of PIMs was reduced from an average of 8 ± 3 PIMs per 1000 orders in 2022 to a rate of 5 ± 3 PIMs per 1000 orders in 2023, as shown in Figure 3.

Therapeutic class analysis revealed substantial decreases in the prescription of antihistamines, muscle relaxants, and tricyclic antidepressants.

**Figure 6:**
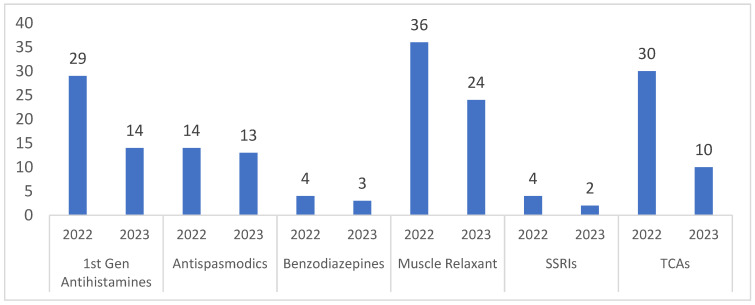
Yearly PIM orders by therapeutic class, RH geriatric OP pharmacy.

Figure 6 shows the annual dispensing pattern of different PIMs in the RH geriatric OP pharmacy from April 2022 to September 2023. A consistent reduction between 9 months in 2022 compared to 9 months during 2023 was observed. In the OP pharmacy, the results show a percentage reduction in TCA of 66.6% (from 30 to 10), 51.7% in antihistamines (from 29 to 14), and 33.3% in muscle relaxants (from 36 to 24).

## 5. Discussion

We found a reduction in potentially inappropriate medications (PIMs) in OPD in the geriatric pharmacy, with about a two-thirds reduction in TCA and a 50% reduction in antihistamine prescriptions. A similar magnitude of decrease in antihistamine prescriptions was seen in dermatology clinics.

### 5.1. Multifaceted Approaches

International efforts to monitor and reduce (PIM) use many interventions, including the use of explicit criterion-based measures, multidisciplinary meetings, and pharmacist-led assessments [11,12]. Applying the explicit tool as guidance in providing education in our study is similar to other international efforts of the World Health Organization (WHO) that aim to enhance knowledge, skills, and awareness of safe medication practices to reduce the likelihood of errors and harm [13]. A systematic review included forty-seven articles that used various interventions to reduce PIM, such as medication reviews, educational strategies, clinical decision support systems, and organizational and multifaceted approaches. The authors found that the most successful intervention in the hospital was medication review (75.0%), while in primary care, educational strategies were the most effective intervention [14].

In the Swedish healthcare system, they applied national quality indicators, which have demonstrated an improvement in the quality of drug prescribing. Between 2006 and 2012, there was a significant reduction in the prescription of drugs that should be avoided in older individuals [15], which is similar to our study; they used the Beers Criteria focusing on medication that should be avoided in older adults, starting with a manual process and providing education and orientation to pharmacy and physician teams.

### 5.2. Integrating with EHRs

Integrating the manual interventional tool in the Electronic Health Record (EHR) system is planned to be the next phase of this study. In U.S. studies, applying a clinical decision support system (CDS) instead of a manual approach reduced PIM prescriptions by 5.2%, which declined by 18.8% upon transitioning the CDS to a new EHR system [16]. This needs special consideration when integrating a CDS within EHRs. It is essential before integrating the tool electronically to consider a multidisciplinary approach, bringing together experts from EHRs in biomedical informatics, web and systems design, geriatric medicine, and geriatric pharmacy to show a significant impact in reducing PIMs. A starting point could be to collate data on patient age, chronic conditions, and medications in the Electronic Health Records (EHRs) using the Health Information Systems Technology Architecture’s (VistA) EMR Web Services [17].

Applying the tool in the EHRs at HMC will improve appropriate prescribing for older adults, as EHR optimization crosses all specialties by default. As shown in our study, reducing PIMs remained challenging for medications initiated from other specialties, such as neurology and psychiatry. Discontinuing or reducing the dose would require a joint decision by geriatrics and other specialties.

### 5.3. Challenges and Future Plan

Some lessons can be learned from the documented challenges, emphasizing the need to improve collaboration and communication between different specialties when any reduction in PIMs is attempted. The clinical intervention documentation shows challenges faced during order modification. Most of them related to PIMs that required discussion with other specialties, such as psychiatry and neurology, where geriatricians were reluctant to discontinue or reduce PIM doses by referring to the original prescriber. At the same time, some patients refused to change or stop their medications, and some prescribers from HMC facilities and primary healthcare centers (PHCCs) were unreachable. In addition, medication management clinics in neurology, pain management, medicine, cardiology, and mental health should be established and activated by having an efficient referral system to geriatric experts and monitoring the time frame of solving medication concerns or ADRs, as doctors cannot override other specialties. Reported challenges in clinical intervention documentation accounted for 70% of the total findings.

Despite the discussion and counseling provided by pharmacists, a substantial proportion (20%) of the total documented interventional findings were related to patients who were unwilling to stop their PIMs. In this study, we found patients may fear change, have concerns about the potential side effects of new medications, or lack understanding of the rationale for changing PIMs. This highlights the considerable effort needed to increase patient awareness and the importance of education and not initiating PIMs without patients’ clear understanding. Healthcare professionals must communicate openly and empathetically with patients to address patient refusal to change PIM orders. Understanding patients’ concerns, beliefs, and preferences regarding their medications can help tailor the approach accordingly. Active listening and addressing patients’ concerns can foster trust and collaboration, enhancing the likelihood of acceptance. A strategy is needed for older patients reluctant to undergo deprescribing [18,19]. Internationally, many initiatives have been implemented to educate older adults about PIM risks and alternative medication benefits and promote informed decision-making while explaining the rationale and potential health improvements to enhance understanding. Utilizing educational materials and visual aids and involving family caregivers aids in patient comprehension and acceptance [20,21]. Incorporating shared decision-making by involving patients in medication management discussions will ensure the patient’s values, preferences, and treatment goals are respected, fostering a sense of ownership and acceptance of the medication changes [22]. Providing gradual medication titration, longitudinal follow-ups, and support in cases where patients are particularly reluctant to change PIM orders can be adopted. Implementing stepwise changes can ease patients into the new treatment regimen, minimizing abrupt changes and potential adverse effects [23].

### 5.4. Performance Quality Indicators

Applying pharmacy monitoring KPIs for process quality measures by reviewing the clinical intervention documentation to evaluate potential facilitators and barriers in the healthcare system provides insights into the challenges of optimizing future interventions to improve medication management practices.

The Swedish National Board of Health and Welfare supports monitoring prescribing patterns by applying quality indicators, which are divided into two groups: drug-specific and diagnosis-specific. Some of these indicators are suitable for analyzing dispensed drugs in large populations, while the drug-specific indicators include medications that should be avoided in older adult populations [24]. We have, therefore, included the specific class of PIMs in our chosen outcomes.

This study highlights the positive impact of using the QTRIM to efficiently reduce PIMs and how the pharmacy monitoring approach and applying quality measures have contributed to this reduction. It emphasizes the need to collaborate with other specialties and services in HMC and PHCCs. It underscores the imperative for adopting comprehensive methodologies aimed at augmenting prescribing practices and fostering enhanced patient-centered care paradigms.

### 5.5. Limitations and Strengths

The scope of our study was confined to outpatient department (OPD) pharmacies, addressing only a segment of medication dispensing practices in HMC. Specifically, this study focused on the OPD pharmacy’s role in assuring prescribers’ awareness and preventing the dispensing of inappropriate medications. The implementation of the tool supports the awareness of the QTRIM among Rumailah Hospital (RH) prescribers, while other prescribers from different PHCCs and other HMC specialties were not included.

Implementing a manual tool may require frequent communication and follow-ups with prescribers to ensure awareness of its usage. The manual tool included 24 items in Phase 1, which is considered a limitation even if it is agreed to be expanded to cover more items in Phase 2. The outcome measure centered on using PIMs, but we did not measure the effect of the intervention on patient clinical outcomes or experience.

Despite the limitations, our study signifies a notable advancement in Potentially Inappropriate Medication (PIM) prescribing within Qatar’s outpatient landscape, especially when applied in Cerner. Our study is the first study in Qatar focused on developing a tool to reduce inappropriate medication prescribing in older adults. The challenges encountered in our study gave a clear understanding of the future initiatives that could be applied to improve the practice. The application of the manual tool will facilitate future electronic integration as a clinical decision support system in the next phase. Integrating the manual tool into the Cerner system will streamline communication and increase the awareness of PIMs among all prescribers and pharmacy staff in different sectors in and outside of HMC. The QTRIM, associated implementation efforts, education, and quality monitoring should be considered to expand the positive outcome to other settings in HMC and PHCCs. This underscores the imperative for adopting comprehensive methodologies to augment prescribing practices and foster enhanced patient-centered care paradigms.

## 6. Conclusions

Implementing an explicit tool (QTRIM) with the multifaceted intervention of providing education and guidance and monitoring implementation performance reduced PIM prescribing in the geriatric and dermatology clinics of HMC, a major healthcare provider in Qatar. This study exemplifies the crucial role of pharmacies in reducing PIMs and monitoring performance improvement. This highlights the potential of expanding the project with collaboration and coordination between all HMC facilities and PHCCs. We identified some challenges that can be addressed in the future by embracing a multifaceted and collaborative approach. Healthcare systems should implement appropriate processes and procedures to ensure the sustained expansion and success of medication safety initiatives like QTRIM, which will contribute to improved patient outcomes and healthcare quality.

## Figures and Tables

**Table 1:** Qatar Tool for Reducing Potentially Inappropriate Medication—QTRIM.

Class	Items	Rationale for Not Prescribing	Potential Alternatives
TCA	Amitriptyline	Strong anticholinergic properties and potential for sedation and orthostatic hypotension, syncope, bradycardia, syndrome of antidiuretic hormone secretion (SIADH), or hyponatremia.	Neuropathic pain: gabapentin, pregabalin, and duloxetine (mainly if depression exists). Depression: SNRIs, SSRIs except paroxetine, and low dose of mirtazapine.
	Imipramine		Migraine and headache prevention; for other migraine prophylaxis, consider targeting other comorbidities (antidepressants, antihypertensives, and anticonvulsants).
	Clomipramine, Trimipramine		
Muscle Relaxants	Orphenadrine	Older adults poorly tolerate most muscle relaxants due to anticholinergic effects caused by some muscle relaxants, risk of sedation, delirium, and an increased risk of fracture. In addition, efficacy is questionable at doses tolerated by geriatric patients.	Non-pharmacological interventions, such as paracetamol; local applications/injections. And, other pain medications according to pain type, location, duration, and intensity.
NSAIDs		Avoid due to increased risk of gastrointestinal bleeding/peptic ulcer disease and acute kidney injury in older adults. Indomethacin is more likely than other NSAIDs to have adverse CNS effects. Of all the NSAIDs, indomethacin has the most damaging effects.	Non-pharmacological interventions, such as paracetamol; LAs, local injections, and other pain medications according to pain type, location, duration, and intensity.
	Ketorolac		If there is no practical alternative, use low-dose selective COXII-NSAIDs (such as celecoxib and etoricoxib) for the shortest period, along with PPI.
	Indomethacin		
First-Generation Antihistamines	Chlorpheniramine, cyproheptadine, and diphenhydramine (oral, hydroxyzine, clemastine, and promethazine	Potent anticholinergic properties, resulting in an increased risk of confusion/delirium, dry mouth, and constipation; use should also be avoided due to reduced clearance with advanced age and tolerance associated with use as a hypnotic.	Allergy: non-sedating, non-anticholinergic antihistamines like desloratadine and levocetirizine. Sleep disturbances: see BZD below. Nausea: treat the cause; consider ondansetron if indicated.
			Dystonia including EPS: diphenhydramine injection.
Insulins	Aspart, glulisine, lispro, and regular in the absence of basal/intermediate insulin for chronic DM management	There is a higher risk of hypoglycemia associated with sliding-scale insulin without improvements in hyperglycemia, regardless of the care setting.	Tailored diabetes management plan considering antidiabetics with low hypoglycemic risk, such as metformin, gliptins, and cardioprotective agents, particularly for cardiac patients (gliflozins and GLP1 agonists). Consider adding basal/intermediate insulin to fast-/short-acting insulin. Consider decreasing the current dose of insulin when starting the new antidiabetics.
SSRIs (Selective Serotonin Reuptake Inhibitors)	Paroxetine	Strong anticholinergic properties and potential for sedation and orthostatic hypotension, falls or fractures, ataxia, impaired psychomotor function, syncope, and cause or exacerbate syndrome of inappropriate antidiuretic hormone secretion or hyponatremia.	Consider non-pharmacological interventions, other SSRIs, SNRIs, and low-dose mirtazapine,3 with appropriate monitoring of falls, ECG, and electrolytes.
Anticholinergics Antiparkinsonians	Procyclidine	Strong anticholinergic properties, and not recommended for the prevention of extrapyramidal symptoms with antipsychotics. In the treatment of Parkinson‘s disease, more effective agents are available.	Parkinson’s: Consider adding or adjusting dopaminergic medications (particularly levodopa/carbidopa if indicated).
	Trihexyphenidyl		Dystonia including EPS: the HMC formulary includes a diphenhydramine injection.
	Benztropine (oral)		
Antispasmodics	Clidinium chlordiazepoxide, diphenoxylate and atropine, and scopolamine (excludes ophthalmic)	Highly anticholinergic properties and uncertain effectiveness as an antispasmodic. Chlordiazepoxide increases the risk of impaired cognition, delirium, falls, and fractures and has a slower metabolism in older adults.	Treating the cause: if an antispasmodic is required, consider a drug with lower anticholinergic properties, such as mebeverine.
Benzodiazepines	Alprazolam	Increased risk of impaired cognition, delirium, falls, fractures, and motor vehicle accidents with benzodiazepine use.	Sleep disturbance: non-pharmacological interventions, such as melatonin, low doses of mirtazapine (7.5–15 mg/d), and low-dose trazodone (25–50 mg/d).
	Temazepam		Anxiety: antidepressants with an anxiolytic profile (SSRIs except paroxetine).
Cardiovascular Medications	Nifedipine immediate release and methyldopa	Nifedipine: the potential to cause hypotension and risk for precipitating myocardial ischemia.	Sustained-release nifedipine or other CCB may be used if indicated; other safer antihypertensive initiation or intensification (such as CBB, and ACEIs/ARBs).
		Methyldopa: high risk of CNS ADRs and risk of bradycardia and orthostatic hypotension.	

**Table 2:** Key performance indicator measures.

Process Measure	Definition	The percentage of PIM orders dispensed from the pharmacy with documented pharmacist intervention.
	Numerator	The number of monthly PIM orders dispensed from the pharmacy with documented pharmacist intervention.
	Denominator	The total number of PIM orders dispensed from the pharmacy to older adults in a calendar month.
Outcome Measure	Definition	The rate of PIMs dispensed from the pharmacy to older adults in a calendar month.
	Numerator	The number of monthly PIM orders dispensed from the pharmacy to older adults in a calendar month multiplied by a standard population.
	Denominator	The total number of orders dispensed by a pharmacy to older adults in a calendar month.

**Table 3:** Baseline characteristics for older adults on PIMs.

Year Total Orders		2022	2023	Total
		N = 220	N = 117	N = 337
Age, Mean (SD)		70 (7.13)	70 (6.90)	70 (7.04)
Sex, n (%)				
	Female	100 (45%)	55 (47%)	155 (46%)
	Male	120 (55%)	62 (53%)	182 (54%)
Dispense Location, n (%)				
	RH Dermatology OP Pharmacy	114 (52%)	63 (54%)	177 (53%)
	RH OP Pharmacy	106 (48%)	54 (46%)	160 (47%)
PIM, n (%)	PIM			
	ALPRAZolam	4 (2%)	3 (3%)	7 (2%)
	Hyoscine N Butyl Bromide	12 (5%)	6 (5%)	18 (5%)
	PARoxetine	2 (1%)	1 (1%)	3 (1%)
	Amitriptyline	29 (13%)	14 (12%)	43 (13%)
	Chlorpheniramine	3 (1%)	2 (2%)	5 (1%)
	ClomiPRAMINE	7 (3%)	1 (1%)	8 (2%)
	Cyproheptadine	1 (0%)	1 (1%)	2 (1%)
	DiphenhydrAMINE/NH4Cl/Na Citrate/menthoL	8 (4%)	0 (0%)	8 (2%)
	HydrOXYzine	106 (48%)	54 (46%)	160 (47%)
	Imipramine	1 (0%)	1 (1%)	2 (1%)
	Paracetamol-orphenadrine450/35	47 (21%)	32 (27%)	79 (23%)
	Promethazine hydrochloride	0 (0%)	2 (2%)	2 (1%)
Race, n (%)				
	Arab	147 (67%)	83 (71%)	230 (68%)
	Asian	43 (20%)	25 (21%)	68 (20%)
	Black	20 (9%)	6 (5%)	26 (8%)
	Persian	5 (2%)	1 (1%)	6 (2%)
	White	5 (2%)	2 (2%)	7 (2%)
Therapeutic Class, n (%)	Therapeutic Class			
	First-Gen Antihistamines	118 (54%)	59 (50%)	177 (53%)
	Antispasmodics	12 (5%)	6 (5%)	18 (5%)
	Benzodiazepines	4 (2%)	3 (3%)	7 (2%)
	Muscle Relaxants	47 (21%)	32 (27%)	79 (23%)
	SSRIs	2 (1%)	1 (1%)	3 (1%)
	TCAs	37 (17%)	16 (14%)	53 (16%)

## Data Availability

The original contributions presented in the study are included in the article/Supplementary Materials, further inquiries can be directed to the corresponding author.

## Supplementary Materials

The following supporting information can be downloaded at: https://www.mdpi.com/article/10.3390/healthcare12121186/s1, Table S1: RH OP “Clinical Intervention Documentation”; Table S2: RH Dermatology OP “Clinical Interventions Documentation”; Table S3: RH OP “Rate of PIMs Rx per 1000 medication orders”; Table S4: RH Dermatology OP “Rate of PIMs Rx per 1000 medication orders”.
