# Supplementary material for: Enhancing Medication Safety through Implementing the Qatar Tool for Reducing Inappropriate Medication (QTRIM) in Ambulatory Older Adults
Source: Healthcare (Basel). 2024 Jun 12;12(12):1186. doi: 10.3390/healthcare12121186 (PMC11202920; doi:10.3390/healthcare12121186)
Supplement: Supplementary file 1 [file healthcare-12-01186-s001.zip › healthcare-3012069-supplementary.pdf]

Supplementary Tables.

Table S1: RH OP "Clinical Intervention Documentation"					
Month	Value	Numerator	Denominator	Goal	Median
Jul-22	50%	5	10	100%	100%
Aug-22	67%	10	15	100%	100%
Sep-22	83%	5	6	100%	100%
Oct-22	50%	1	2	100%	100%
Nov-22	60%	3	5	100%	100%
Dec-22	100%	6	6	100%	100%
Jan-23	100%	4	4	100%	100%
Feb-23	100%	3	3	100%	100%
Mar-23	100%	4	4	100%	100%
Apr-23	100%	4	4	100%	100%
May-23	100%	6	6	100%	100%
Jun-23	100%	5	5	100%	100%
Jul-23	100%	4	4	100%	100%
Aug-23	100%	5	5	100%	100%
Sep-23	100%	3	3	100%	100%
Oct-23	100%	6	6	100%	100%
Nov-23	100%	4	4	100%	100%
Dec-23	100%	3	3	100%	100%

Table S2: RH Dermatology OP "Clinical Interventions Documentation"					
Month	Value	Numerator	Denominator	Goal	Median
Jul-22	75%	3	4	100%	100%
Aug-22	73%	8	11	100%	100%
Sep-22	100%	8	8	100%	100%
Oct-22	100%	12	12	100%	100%
Nov-22	90%	9	10	100%	100%
Dec-22	100%	10	10	100%	100%
Jan-23	100%	16	16	100%	100%
Feb-23	100%	7	7	100%	100%
Mar-23	100%	9	9	100%	100%
Apr-23	100%	1	1	100%	100%
May-23	100%	4	4	100%	100%
Jun-23	100%	2	2	100%	100%
Jul-23	100%	9	9	100%	100%
Aug-23	100%	11	11	100%	100%
Sep-23	100%	8	8	100%	100%

Oct-23	100%	8	8	100%	100%
Nov-23	100%	7	7	100%	100%
Dec-23	100%	5	5	100%	100%

Table S3: RH OP "Rate of PIMs Rx per 1000 medication orders"

Month	Value	Numerator	Denominator	Goal	Median
Apr-22	1.1	7	6309	0	0.9
May-22	1.2	6	5142	0	0.9
Jun-22	1.0	5	5207	0	0.9
Jul-22	2.2	10	4597	0	0.9
Aug-22	2.5	15	5919	0	0.9
Sep-22	1.1	6	5558	0	0.9
Oct-22	0.3	2	6312	0	0.9
Nov-22	0.8	5	6356	0	0.9
Dec-22	1.0	6	5780	0	0.9
Jan-23	0.6	4	6491	0	0.9
Feb-23	0.5	3	5615	0	0.9
Mar-23	0.6	4	6708	0	0.9
Apr-23	0.9	4	4322	0	0.9
May-23	0.8	6	7107	0	0.9
Jun-23	0.9	5	5718	0	0.9
Jul-23	0.7	4	5413	0	0.9
Aug-23	0.9	5	5585	0	0.9
Sep-23	0.7	3	4446	0	0.9
Oct-23	1.1	6	5588	0	0.9
Nov-23	0.7	4	5627	0	0.9
Dec-23	0.5	3	5945	0	0.9

Table S4: RH Dermatology OP "Rate of PIMs Rx per 1000 medication orders"

Month	Value	Numerator	Denominator	Goal	Median
Apr-22	12.3	14	1138	0	7.1
May-22	9.6	10	1042	0	7.1
Jun-22	11.2	16	1426	0	7.1
Jul-22	4.8	4	834	0	7.1
Aug-22	7.8	11	1410	0	7.1
Sep-22	7.5	8	1065	0	7.1
Oct-22	6.9	12	1730	0	7.1
Nov-22	6.8	10	1473	0	7.1
Dec-22	8.2	10	1220	0	7.1
Jan-23	9.4	16	1698	0	7.1
Feb-23	4.8	7	1447	0	7.1

Mar-23	5.8	9	1543	0	7.1
Apr-23	1.1	1	896	0	7.1
May-23	2.0	4	1954	0	7.1
Jun-23	1.7	2	1189	0	7.1
Jul-23	7.1	9	1272	0	7.1
Aug-23	7.2	11	1535	0	7.1
Sep-23	6.0	8	1344	0	7.1
Oct-23	6.2	8	1282	0	7.1
Nov-23	3.9	7	1775	0	7.1
Dec-23	4.0	5	1265	0	7.1
